# Investigating the Interaction of Ascorbic Acid with Anthocyanins and Pyranoanthocyanins

**DOI:** 10.3390/molecules23040744

**Published:** 2018-03-23

**Authors:** Jacob E. Farr, M. Monica Giusti

**Affiliations:** Department of Food Science and Technology, The Ohio State University, 2015 Fyffe Ct., Columbus, OH 43210-1007, USA; farr.39@osu.edu

**Keywords:** anthocyanins, pyranoanthocyanins, ascorbic acid, bleaching, condensation

## Abstract

Juices colored by anthocyanins experience color loss related to fortification with ascorbic acid (AA), thought to be the result of condensation at Carbon-4 of anthocyanins. To further understand this mechanism, pyranoanthocyanins, having a fourth-ring covalently occupying Carbon-4, were synthesized to compare its reactivity with AA against that of anthocyanins. Pyranoanthocyanins were synthesized by combining chokeberry anthocyanins with pyruvic acid. AA (250–1000 mg/L) was added to either chokeberry extract, cyanidin-3-galactoside, or 5-Carboxypyranocyanidin-3-galactoside. Samples were stored in the dark for 5 days at 25 °C and spectra (380–700 nm), color (CIE-L*c*h*), and composition changes (HPLC-MS/MS) were monitored. Extensive bleaching occurred for cyanidin-3-galactoside and chokeberry colored solutions, with a decrease in half-lives from 22.8 to 0.3 days for Cyanidin-3-galactoside when 1000 mg/L AA was added. 5-Carboxypyranocyanidin-3-galactoside solution better maintained color with limited loss in absorbance, due to the formation of colored degradation products (λ_vis-max_ = 477 to 487 nm), and half-life decrease from 40.8 to 2.7 days, an 8–13-fold improvement compared to anthocyanins. This suggested alternative sites of reactivity with AA. Carbon-4 may be the preferred site for AA-pigment interactions, but it was not the only location. With Carbon-4 blocked, 5-Carboxypyranocyanidin-3-galactoside reacted with AA to form new pigments and reduce bleaching.

## 1. Introduction

Consumers commonly use color to make assessments on acceptance and liking, implied flavor, safety, and overall quality of food products [1]. Synthetic colorants have been used to correct the natural variation of food items, mask imperfections, as well as offer alternative product identities [1]. The innate stability of synthetic colorants over natural pigments has been a driver for their selection in coloring food products. Recently, this trend has begun to reverse as consumers have expressed concerns over the safety of synthetic colorants and preference for colorants from natural sources [2,3]. Anthocyanins are widely viewed as a natural alternative due to their wide spectrum of hue expression; however, their application has been limited due to stability [4].

Anthocyanins (ACN) are a class of water-soluble polyphenols found in many fruits and vegetables. Their color properties are greatly influenced by the substitution patterns on the aglycone structure as well as pH environment [4]. Warm hues including reds are observed at low pH but shift expression to vibrant purple-blues in more alkaline conditions. Their stability is influenced by many factors including pH, heat, enzymes, light, as well as certain bleaching agents including sulfites, hydrogen peroxide, and vitamin C (ascorbic acid, AA) [5]. The latter is of significance for the food industry, with widespread use of AA as both a fortifying agent as well as an antioxidant in many food and beverage systems [6].

**Figure 1:**
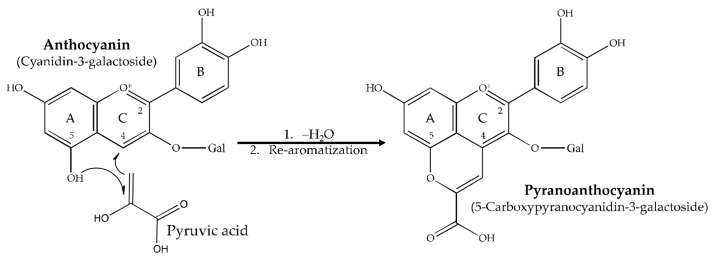
Formation of pyranoanthocyanin from cyanidin and pyruvic acid by heterocyclic addition.

It has long been known that the presence of AA in anthocyanin-colored solutions can accelerate degradation and loss of color [7]. Bisulfites, hydrogen peroxide, and ascorbic acid are electrophilic compounds and are thought to attack the same nucleophilic sites of the anthocyanin. For ascorbic acid, it has been postulated to cause mutual and irreversible destruction of both the pigment and micronutrients [8]. This is differentiated from bisulfite bleaching, which is reversible and pH-dependent [9]. This presents a major hurdle for the food industry to use ACN-based colorants, specifically in juices and beverages which are often fortified with vitamin C. Previous research [10,11] has proposed that anthocyanin bleaching is the result of the condensation of ascorbic acid, as well as other bleaching agents, at Carbon-4 (C4) of the anthocyanin (Figure 1), as this site is the most susceptible to electrophilic attack. However, there is also NMR evidence suggesting alternative sites of bisulfite addition such as Carbon-2 (C2) [12]. The proposed condensation is thought to result in the loss of conjugation in the C-ring, therefore lacking the original color expression of the pigment.

Previous work has found that anthocyanins with both 3- and 5-substitions increase pigment stability against ascorbic acid compared to just 3-substitution, likely a result of further restricting access to C4 in between. Viguera and Bridle reported that Malvidin-3,5-diglucoside experienced slower color loss as compared to Malvidin-3-glucoside. The same authors reported that direct substitution of the C4 with phenyl and methyl groups enhanced their stability against ascorbic acid color loss versus typical –H substitution [11]. Copigmentation of grape anthocyanins with rosemary polyphenols has also been shown to have a protective effect on the pigment; it is possible that the π-π interaction can limit accessibility to the chromophore [13]. Another means by which the interaction between anthocyanins and ascorbic acid could be investigated is by evaluation and comparison to pyranoanthocyanins.

Pyranoanthocyanins (Figure 1) are formed by anthocyanins undergoing heterocyclic addition of a polar carboxyl-containing compound such as pyruvic acid, acetaldehyde, or catechins, which are often byproducts of yeast fermentation [14]. This results in the formation of a fourth ring (D) that covalently occupies C4 and C5 of the pigment. Pyranoanthocyanins (PACN) are found in aged wines and have been reported in red onions and strawberries [14,15,16]. Previous research on pyranoanthocyanin stability has shown their enhanced resistance to bisulfite bleaching [17,18,19] but not to ascorbic acid. Carboxy-pyranoanthocyanins, resulting from the reaction of Malvidin-3-glucoside from grape with pyruvic acid, showed enhanced stability against bisulfite bleaching (up to 250 ppm) compared to anthocyanins [17]. Oligomeric pyranoanthocyanins were shown to exhibit complete resistance to bisulfite bleaching (up to 250 ppm) for 2 days [17]. It is possible that the oligomeric structures also block other potential sites of reaction such as C2. Acetyl-pyranoanthocyanins, synthesized with acetaldehyde, have been shown to not only overcome bisulfite bleaching, but were also reported to experience a hyperchromic shift in response to bisulfite at up to 200 ppm, an unexpected response to a common bleaching agent [19].

The objective of this study was to compare the reactivity of anthocyanins and pyranoanthocyanins upon the addition of AA. If anthocyanin bleaching with AA occurs only at site C4, pyranoanthocyanins, with C4 unavailable, would not undergo bleaching. Furthermore, no changes would occur on the PACN pigments as a results of AA addition. Our hypothesis was that C4 is not the only site of reactivity. Comparison of the reactivity of these pigments in response to ascorbic acid will aid in further understanding the deleterious interaction of this micronutrient and pigment and could allow for the better selection of anthocyanin sources in applications with ascorbic acid.

## 2. Results and Discussion

### 2.1. UV-Vis Spectrophotometry of Solutions

**Figure 2:**
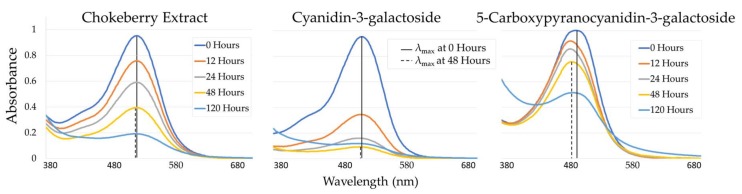
Spectral absorbance changes in response to 500 mg/L ascorbic acid (AA) for chokeberry, cyanidin-3-galactoside, and 5-Carboxypyranocyanidin-3-galactoside colored solutions over a 5-day period.

Anthocyanins degraded quickly in the presence of ascorbic acid, as seen in Figure 2. Chokeberry extract, containing an ACN profile which is ~70% Cyanidin-3-galactoside and with anthocyanins representing ~35% of the total area under the curve (AUC) in the max plot, showed greater resistance to bleaching compared to the purified Cyanidin-3-galactoside. This is likely the result of other chokeberry phenols playing a protective role against AA-induced degradation. Copigmentation as well as antioxidant capacity have both been demonstrated as a way by which additional polyphenols protect anthocyanins [20,21,22]. Other phenolic constituents of chokeberry include procyanidins, quercetin derivatives, epicatechin, and chlorogenic acid [23]. PACN (5-Carboxypyranocyanidin-3-galactoside) derived from Cyanidin-3-galactoside showed the least change in absorbance over time. Covalently occupying C4 in 5-Carboxypyranocyanidin-3-galactoside was thought to result in less change in absorbance as compared to Cyanidin-3-galactoside and chokeberry, similar to other reports of bleaching of pyranoanthocyanins observed with bisulfites [18]. All pigment solutions with ascorbic acid experienced signficant changes in maximum absorbance over time with *p*-values of less than 0.01. As AA levels increased, the loss in absorbance for each pigment over time also increased, revealing a dose-dependent effect of AA. For the 500 mg/L AA treatments over a 5-day period, 5-Carboxypyranocyanidin-3-galactoside saw a reduction of 38% reduction in maximum absorbance, chokeberry a 79% reduction, and Cyandin-3-galactoside an 88% reduction.

Changes in λ_vis-max_ were also observed over the 5-day period. For the 500 mg/L AA level, the following hypsochromic changes in λ_vis-max_ occurred: chokeberry, 512 to 511 nm; cyanidin-3-galactoside, 511 to 509 nm, 5-Carboxypyranocyanidin-3-galactoside, 491 to 484 nm. These shifts in λ_vis-max_ are reflected in Figure 2. The change for 5-Carboxypyranocyanidin-3-galactoside correlated with the newly developed peaks discovered during HPLC analysis, later discussed, and resulted in the solution being more orange-red. Hypsochromic changes on ACN (chokeberry extract and cyanidin-3-galactoside) λ_vis-max_ observed over the 5 days of the AA treatment were less than 5 nm, regardless of the levels of AA, and were more likely attributed to pigment degradation. The PACN experienced hypsochromic shifts as large as 10 nm with less loss in maximum absorbance, with shifts becoming more pronounced as AA levels increased.

### 2.2. Kinetics of Degradation

Degradation kinetics were evaluated in terms of change in maximum absorbance of the solutions at the original λ_vis-max_ over time. Bleaching has been previously reported as a first-order reaction, typical of ACN degradation, and was modeled as such in determining the reaction rate and half-life [24,25]. The decrease in maximum absorbance correlated with an increase in lightness (L*) as well as the decrease in chroma (c*) for all pigments. The reduction in maximum absorbance closely followed first-order kinetics for all treatments with an R^2^ higher than 0.94 for control treatments and 0.96 for samples with added ascorbic acid. The kinetics results for each pigment and AA level can be found in Table 1. Without ascorbic acid, 5-Carboxypyranocyanidin-3-galactoside had the greatest half-life (978 h), followed by chokeberry extract (858 h), and then Cyanidin-3-galactoside (546 h). Addition of ascorbic acid dramatically reduced half-lifes for all pigments. With 1000 mg/L AA added, the 5-Carboxypyranocyanidin-3-galactoside half-life was 64 h; chokeberry extract had a half-life of 24 h; and Cyanidin-3-galactoside had a half-life of 8 h, seen in Table 1.

This order of stability was also exhibited across all AA levels. The enhanced stability and extension of half-life for 5-Carboxypyranocyanidin-3-galactoside was more evident upon the addition of ascorbic acid. Pyranoanthocyanins exhibited a half-life 8–13x higher than Cyanidin-3-galactoside in the presence of AA. Kinetics data supports the hypothesis that C4 is likely the primary or preferred site for anthocyanin-ascorbic acid interaction. It also reveals that the pyranoanthocyanin was still susceptible to ascorbic acid through alternative mechanisms or sites due to the half-life lowering in response to AA compared to its control.

**Figure 3:**
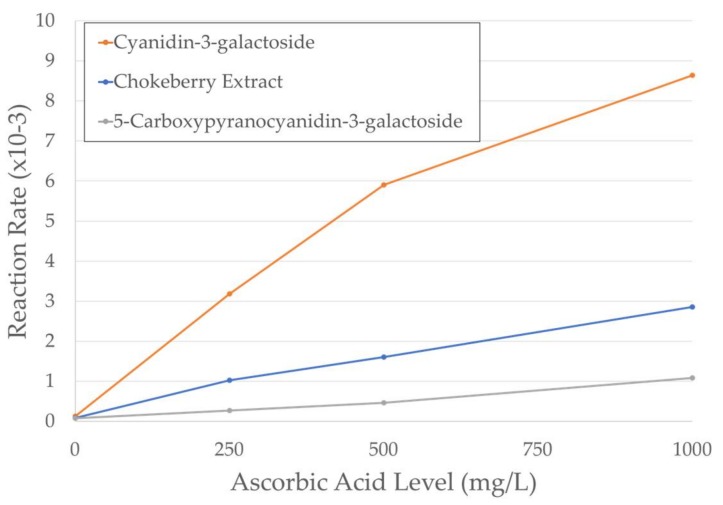
Reaction rates of 5-Carboxypyranocyanidin-3-galactoside, Cyanidin-3-galactoside, and chokeberry plotted against ascorbic acid level (0–1000 mg/L). Calculations are based on the changes in absorbance at the λ_vis-max_ of the solution over time.

The relationship between ascorbic acid level and the reaction rates was additionally assessed to observe how each of these pigments responds to ascorbic acid addition. A linear relationship was found and can be seen in Figure 3. The R^2^ values for these pigments at varying AA levels are the following: chokeberry extract, 0.99; Cyanidin-3-galactoside, 0.96; and 5-carboxypyranocyanidin-3-galactoside, 0.98. Linearity is lost to some degree for Cyanidin-3-galactoside with 1000 mg/L AA addition. The slope could be effectively regarded as how responsive (or deleterious) the change in pigment solution maximum absorbance is upon AA addition, with a higher slope indicating greater susceptibility to AA. The slope of Cyanidin-3-galactoside was 3.1× that of chokeberry extract and, in comparison to 5-carboxypyranocyanidin-3-galactoside, cyanidin-3-galactoside was 8.2× and chokeberry extract 2.7× more suspectible.

### 2.3. Colorimetry

#### 2.3.1. Lightness

**Figure 4:**
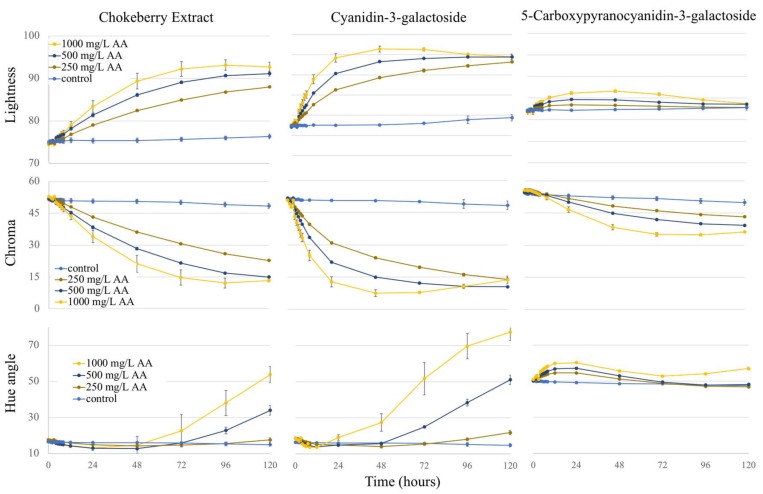
Colorimetric changes (CIE-L*c*h*) of change of solutions colored with chokeberry, cyanidin-3-galactoside, and 5-Carboxypyranocyanidin-3-galactoside from day 0 to day 5 for all AA levels over time. Error bars represent standard deviation.

Rapid color loss and extensive bleaching of pigments can be seen with CIE lightness (L*) in Figure 4. Within 48 h exposed to 1000 mg/L AA, L* increased from 77.2 to 96.4 (∆19.2) for Cyanidin-3-galactoside; chokeberry, 74.4 to 89.4 (∆15.0); and 5-Carboxypyranocyanidin-3-galactoside, 81.7 to 86.6 (∆4.9). The presence of AA resulted in higher lightness over time, and this was dose-dependent. Pyranoanthocyanins showed the least change in L* in reponse to AA, and Cyanidin-3-galactoside the greatest. An increase in L* represents a lighter color expression and was most evident for chokeberry and cyanidin-3-galactoside.

#### 2.3.2. Chroma

Pigment levels were standardized by absorbance at their respective λ_vis-max_; therefore, chroma values were in close agreement at day 0. Chroma, being a measure of color intensity, is useful for determining the extent of bleaching and is reported in Figure 4. The PACN had less change in chroma compared to Cyanidin-3-galactoside and chokeberry. Chroma decreased with increasing AA levels with the exception of Cyanidin-3-galactoside after 48 h, likely the result of ascorbic acid and pigment browning playing a larger role at those times. The changes in chroma in reponse to AA addition followed: Cyanidin-3-galactoside > chokeberry > 5-Carboxypyranocyanidin-3-galactoside. All pigments (including controls) experienced a significant change in chroma over 5 days with *p*-values of less than 0.001.

#### 2.3.3. Hue Angle

Synthesis of pyranoanthocyanins results in a pigment with a lower λ_vis-max_ and a higher hue angle compared to the respective anthocyanin, having a more orange-red color expression as compared to the red color of ACN. This was clearly observed on the initial hue angle values of 5-Carboxypyranocyanidin-3-galactoside (50°), a more orange-red hue than Cyanidin-3-galactoside (16.6°), with a more red color (Figure 4). While the initial hue angle for chokeberry and Cyandin-3-galactoside started much lower and more red (<20°) than the PACN, the reaction between ACN-AA resulted in a dramatic color shift towards a yellow coloration. For the 1000 mg/L AA level, large increases in hue angle were observed from day 0 to 5 for chokeberry (17.6° to 53.8°) and cyanidin-3-galactoside (18.7° to 77.5°), while the hue angle change was much smaller for 5-Carboxypyranocyanidin-3-galactoside, changing from 51.0° to 57.1°. Changes in hue angle in the presence of AA were dose-dependent. The rapid increase in hue angle for cyanidin-3-galactoside and chokeberry was likely the result of pigment degradation and fading, whereas for 5-Carboxypyranocyanidin-3-galactoside, which better retained original chroma and lightness parameters, new pigment formation may explain hue angle changes.

#### 2.3.4. Total Color Change (ΔE)

Total color changes (ΔE) were calculated as the color change from day 0 to 5 for each respective treatment, and are presented in Table 2. Without AA, chokeberry had the smallest ΔE, followed by 5-Carboxypyranocyanidin-3-galactoside and Cyanidin-3-galactoside. Other phenolics present in chokeberry could have enhanced color retention and stability through mechanisms such as copigmentation or radical scavenging by additional phenolics, which would not have been possible with isolated 5-Carboxypyranocyanidin-3-galactoside and cyanidin-3-galactoside. This possible explanation could be supported by previous work, where an abundance of other polyphenols has been reported in chokeberry [23]. The chokeberry fruit has previously been reported to have 89 mg/kg of quercetin, which further supports why greater stability was observed for the chokeberry treatment as compared to purified Cyanidin-3-galactoside [26]. For all levels of AA addition, chokeberry and Cyanidin-3-galactoside had a ΔE greater than 29. However, 5-Carboxypyranocyanidin-3-galactoside with AA exhibited a signficiantly smaller color shift with values of ΔE ranging from 8.7 to 10.9. This three-fold reduction in ΔE resulted in an overall better retention of color in response to AA addition.

### 2.4. HPLC and MS/MS Evaluation

**Figure 5:**
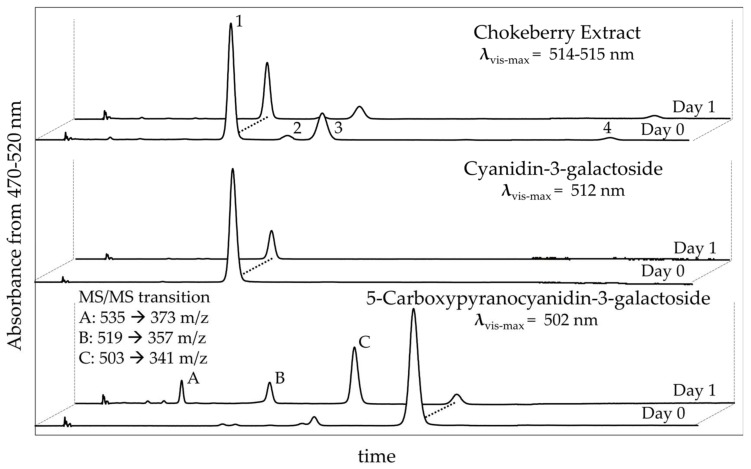
HPLC profiles (470–520 nm) for 5-Carboxypyranocyanidin-3-galactoside, Cyanidin-3-galactoside, and chokeberry with 1000 mg/L ascorbic acid added on days 0 and 1. MS/MS transition reported for the newly formed peaks resulting between 5-Carboxypyranocyanidin-3-galactoside and ascorbic acid interaction.

To determine the relationship between spectral and color changes with changes in pigment composition, HPLC analysis was utilized. The initial (day 0) HPLC profile for chokeberry extract revealed the following anthocyanin profile seen in Figure 5: Cyanidin-3-galactoside (Peak #1, 70%), Cyanidin-3-glucoside (Peak #2, 3%), Cyanidin-3-arabinoside (Peak #3, 23%), and Cyanidin-3-xyloside (Peak #4, 4%), in line with the expected profile [23]. Anthocyanins contributed to 35% of the total AUC in the max plot (260–700 nm), mainly due to the presence of other polyphenols. The largest non-anthocyanin peak with a λ_vis-max_ of 322 nm was likely chlorogenic acid, as it is reported to be present in the berry [23]. After 1 day of exposure to 1 g/L AA, all anthocyanins in the chokeberry extract decreased by 65%. The loss of individual pigments ranged from 64–68%, revealing similar degradation kinetics for all pigments present. This is not surprising considering they are all cyanidin-3-monosaccharides. By day 5, only 4% of the original pigments in the chokeberry extract had survived. The behavior of Cyanidin-3-galactoside was similar to that of chokeberry extract but was thought to experience more rapid bleaching due to the absence of other polyphenols, imparting a protective effect. The isolated anthocyanin accounted for 92% of the AUC in the day 0 maxplot. By day 1, Cyanidin-3-galactoside was reduced by 91% and day 5, >99%.

For the pyranoanthocyanin, the isolated structure accounted for 94% of the AUC from the maxplot. By day 1, this peak was reduced 93% and 99% by day 5; however, unlike the anthocyanins, where the pigments degraded into colorless forms, the PACN-AA interaction resulted in the development of new peaks in the visible range, labeled A, B, and C in Figure 5. Peak A appears to be entirely newly formed in response to AA and was not present in either the control or PACN + AA day 0 treatment. Peaks B and C were present at low levels in both the control and AA treatment at day 0 and could be colored degradation products. It appears that AA promotes the formation of these two compounds with peak B having 3.7× the AUC and peak C 15.9× AUC (470–520 nm) by day 1 as compared to the day 0 control treatment. These newly formed peaks also corroborate the spectra changes observed using the plate reader. The newly formed peaks had the following λ_vis-max_: A, 487 nm; B, 486 nm; and C, 477 nm. The formation of these new compounds aligned with both colorimetric data (increase in hue angle) and the spectral shift (hypsochromic response) that was observed with the solutions in response to AA. The formation of the new peaks is likely how the pyranoanthocyanin solution better maintained original color expression even with the rapid loss of the parent compound. The formation of three new chromophores between the PACN-AA interaction could be the result of several different phenomena, and additional experiments were performed to include MS/MS data of the new structures.

For the three peaks produced after AA addition, it is possible that these are the result of interaction with PACN at alternative sites (not C2 or C4). With the addition of a fourth ring, ascorbic acid could have reacted with the D-ring substitution (carboxylic acid group) and produced colored byproducts. It has previously been reported that acetyl pyranoanthocyanins experience both a hyperchromic and hypsochromic shift in response to up to 200 ppm sulfites, and it was proposed this was the result of sulfite covalent linkage at the acetyl group in the D-ring, enhancing the molar absorptivity [19].

The MS/MS data generated provided valuable insight to the structures of the newly formed compounds. Peak A was the only structure that was not present in trace amounts in the control treatment. A positive ion scan revealed a parent *m*/*z* of 535. This was +18 mass units (m.u.) compared to 5-carboxypyranocyanidin-3-galactoside. A followup product ion scan revealed a daughter ion of 373 *m*/*z*, a transition of −162 m.u. from the parent ion. This is a commonly reported transition for glycosylated molecules and matches the loss of galactose from the structure [27]. This suggests that the aglycone structure is being modified and not the sugar substitution. With a parent *m*/*z* of 535, direct condensation of ascorbic or dehydroascorbic was ruled out. Ascorbic acid degradation byproducts were also considered. Ascorbic acid has previously been reported as being catalyzed by trace levels of metal (1 μM) to form hydrogen peroxide [28,29,30]. Ascorbic acid could form hydrogen peroxide which then could react with the pyranoanthocyanin, but the typical result would involve loss of color, and we would expect a different mass transition. Stebbins et al. [31] reported the formation of 6-hydroxy-cyanidin-3-glucoside from cyanidin-3-glucoside and ascorbic acid, the transitional *m*/*z* being +16 mass units. A different mechanism probably occurs, possibly related to the condensation of the pyranoanthocynin with other AA degradation products, and perhaps even the rearomatization of the molecule.

Peak B, under positive ionization, had a parent *m*/*z* of 519, and the respective product ion scan revealed a daughter ion at 357 mass units. Peak C had a parent *m*/*z* of 503 and the product ion scan revealed a daughter with 341 mass units The shifts for all structures was −162 m.u. from parent to daughter ion, which was again attributed to the loss of galactose. It is important to point out that the *m*/*z* of peak C was lower than that of our starting pyranoanthocyanin (5-carboxypyranocyanidin-3-galactoside, *m*/*z* 517), with a loss of 14 units. Elucidation of these chemical structures will require NMR work, currently underway.

Of the three new compounds, the structural modification induced by ascorbic acid addtion was isolated to the aglycone. Interestingly, single ion monitoring for possible direct condensation products of ascorbic or dehydroascorbic acid with 5-carboxypyranocyanidin-3-galactoside (675 and 673 *m*/*z*, respectively) did not appear in the chromatogram. This suggests that the newly formed peaks are not the result of the direct condensation of ascorbic and dehydroascorbic acids with the pyranoanthocyanin.

To further test whether the formation of the new peaks was in response to hydrogen peroxide formed as a byproduct of ascorbic acid degradation, the treatment was repeated except with hydrogen peroxide in place of ascorbic acid. The sample was monitored with 0-, 8-, and 24-h injection times and and only revealed a reduction in the original pyranoanthocyanin. Surprisingly, the three new peaks formed in response to ascorbic acid were not formed in response to direct H_2_O_2_ addition. No MS/MS transitions observed with PACN + AA were found by the addition of H_2_O_2_. With the three peaks absent in both the PDA and MS chromatogram, this theory was rejected. It was thought that ascorbic acid was degrading and that byproducts other than H_2_O_2_ were reacting with the pyranoanthocyanin.

## 3. Materials and Methods

### 3.1. Materials

Powdered chokeberry fruit was provided by Artemis Inc. (Fort Wayne, IN, USA). Lab grade pyruvic acid used for the synthesis of pyranoanthocyanins was purchased from Sigma Aldrich (St. Louis, MO, USA). Analytical grade ascorbic acid (99% l-ascorbic acid) was purchased from Sigma Aldrich. HPLC grade acetonitrile and water were obtained from Fisher Scientific (Hampton, NH, USA) and HPLC grade formic acid from Sigma Aldrich.

### 3.2. Methods

#### 3.2.1. Anthocyanin Semi-Purification (SPE)

Chokeberry powder was mixed with water acidified with 0.01% HCl prior to purification. The solution was loaded onto a Waters Sep-pak C18 cartridge for solid phase extraction (SPE). The column was then washed with acidified water (0.01% HCl) to remove sugars and acids, followed by washing with ethyl acetate for the removal of the more non-polar phenolics. Pigments were recovered from the cartridge with methanol acidified with 0.01% HCl, and the solvent was removed by rotary evaporation (40 °C, under vaccuum). Pigments were then solubilized and stored in acidified water for future use. This was the only preparatory step for chokeberry treatments.

#### 3.2.2. Pyranoanthocyanin Synthesis

Pyrananthocyanins were synthesized from the semi-purified chokeberry by the addition of pyruvic acid. The extract (1000 μM cyanidin-3-glucoside equivalent) was added to a pH 2.6 citrate buffer that had 0.1% potassium sorbate and 0.1% sodium benzoate to prevent molding. A molar ratio of 1:50 (ACN: pyruvic acid) was followed as previously described [17]. The prepared anthocyanin pyruvic acid solution was stored in an incubator in the dark at 35 °C for 10 days (Isotemp, Fisher Scientific, Waltham, MA, USA). After the incubation period ended, cyanidin-3-galactoside and 5-Carboxypyranocyanidin-3-galactoside, the resulting pyranoanthocyanins from cyanidin-3-galactoside and pyruvic acid, were isolated from the solution using semipreparatory HPLC.

#### 3.2.3. Anthocyanin and Pyranoanthocyanin Purification

A reverse phase HPLC system composed of the following modules was used: LC-6AD pumps, CBM-20A communication module, SIL-20A HT autosampler, CTO-20A column oven, and SPD-M20A Photodiode Array detector (Shimadzu, Columbia, MD, USA). The reverse-phase column selected was a 250 × 21.2 mm Luna pentafluorophenyl column with 5 µm particle size and 100 Å pore size (Phenomenex, Torrance, CA, USA). Samples were filtered prior to injection with a Phenex RC 0.45 µm, 15 mm membrane syringe filter (Phenomenex, Torrance, CA, USA). With a flow rate of 10 mL/min and a run time of 30 min, peaks were separated and collected. An isocratic system with the following solvents were used: 11:89 (Solvent A: Solvent B *v*/*v*) with Solvent A being 4.5% formic acid in HPLC grade water and Solvent B was HPLC grade acetonitrile. Elution of peaks was monitored at 500 nm. Peaks were manually collected. The two collected peaks were diluted with distilled water and again subjected to SPE semi-purification to remove formic acid and acetonitrile. Rotary evaporation was used to remove methanol (40 °C, under vaccuum), and the pigments were stored in 0.01% HCl in acidified water.

#### 3.2.4. Anthocyanin and Pyranoanthocyanin Purity

Prior to experimentation, pigments were evaluated for purity by using an analytical HPLC only different from the previously listed one by the use of different pumps (LC-20AD, Shimadzu). Purified pigments were filtered using Phenex RC 0.45-µm membranes. A binary system with 1 mL/min flow rate was used: solvent consisted of 4.5% formic acid in HPLC grade water and solvent B consisted of HPLC grade acetonitrile. The gradient began with an isocratic flow of 6% solvent B for 17 min (elution of primary anthocyanins), increasing to 15% solvent B by 45 min (elution of primary pyranoanthocyanin), and to 40% solvent B by 50 min (wash). A 10-µL injection volume was loaded onto a Phenomenex Kinetix 5-µm EVO C18 100 A. 150 × 4.6 mm column with a Phenomenex Ultra UHPLC EVO C18 guard cartridge attached. Purity was expressed in terms of % peak area of targeted pigment as compared to the total area of all peaks present in the max plot (260–700 nm). The isolate of 5-Carboxypyranocyanidin-3-galactoside accounted for 94% of the overall area under the curve (AUC), cyanidin-3-galactoside isolate was 92% AUC, while chokeberry ACN purity was 35% AUC. Chokeberry ACN likely contained other phenols present in the source material.

#### 3.2.5. Sample Preparation

The semi-purified chokeberry extract, the isolated cyanidin-3-galactoside, and the purified 5-Carboxypyranocyanidin-3-galactoside were diluted in pH 3.0 citrate buffer (0.1 M adjusted with HCl) until an absorbance of 1.0 at their respective λ_vis-max_ was reached. Levels of AA of 250, 500, and 1000 mg/L were added using a concentrated ascorbic acid stock solution, and a control consisting of each pigment with the absence of AA was maintained. All samples were brought to the same final volume with additional citrate buffer. The pH of all samples were evaluted using a S220 SevenCompact pH meter (Mettler Toledo, Columbus, OH, USA) and were found to have a pH of 3.0 ± 0.05. Samples were stored in the dark at 25 °C in an incubator (listed in Section 3.2.2). UV-Vis spectrophotometry, colorimetry, and HPLC analyses were conducted over a 5-day period following the addition of ascorbic acid. UV-Vis spectral data was collected every hour for the first 8 h, and then at 12 h, and daily from that point on for 5 days. Spectra and color were eveluated with this data. HPLC analyses were conducted on days 0, 1, 3, and 5. All treatments were run in triplicate.

#### 3.2.6. UV-Vis Spectrophotometry of Samples

A SpectraMax 190 Microplate Reader (Molecular Devices, Sunnyvale, CA, USA) with a 96-well plate (poly-d-lysine coated polystyrene) were used for the evaluation of absorbance from 380–700 nm, at 1-nm intervals. Aliquots (200 µL) of samples were loaded into individual wells, and a blank consisted of the same citrate buffer used.

#### 3.2.7. Color Analyses of Samples

Using UV-Vis spectral data (380–700 nm, 1-nm intervals) in combination with software (*ColorBySpectra*) written for color conversion, absorbance data was translated to the CIE (Commission internationale de l’éclairage) L*c*h color space [32]. The calculations for CIE-L*c*h* implemented by the software used the following conditions: D65 standard illuminant, regular transmission, and a 10° observer angle function [33]. Color data is reported as L* (lightness), c* (chroma), and h* (hue angle).

#### 3.2.8. HPLC Monitoring of Samples

Prepared solutions were monitored to determine the formation of potential degradation products or profile changes. Using the analytical HPLC system and conditions previously described (Section 3.2.4), chromatograms were monitored with the max plot (260–700 nm), 490 nm (near λ_vis-max_ of 5-Carboxypyranocyanidin-3-galactoside), and 520 nm (near λ_vis-max_ of cyanidin-3-galactoside). A max plot of 470–520 nm was later added to standardize changes in the AUC for all three pigments and their degradation products.

#### 3.2.9. MS/MS Evaluation of Pigments

With the development of newly formed peaks for the pyranoanthocyanin, tandem mass spectrometry (MS/MS) was performed to obtain additional structural information on the novel structures. The same HPLC conditions and column mentioned in Section 3.2.4 were used on a uHPLC (iNexera) system coupled to a tandem MS unit (LCMS 8040) (Shimadzu). Electrospray ionization was used with the following conditions: 1.5 L/min nebulizing gas flow, 230 °C desolvation line temperature, 200 °C heat block temperature, and 15 L/min drying gas flow. Two total ion scans (Q3) were performed, both in positive and negative mode from 100–1500 mass unit with an event time of 0.1 s. Based on the scan results, the following events were added (positive mode) and the sample reran with secondary collisions in argon gas: product ion scan, 535 *m*/*z*; product ion scan, 519 *m*/*z*; product ion scan, 503 *m*/*z*; and precursor ion scan, 355 *m*/*z*. These secondary collision events all had an event time of 0.1 s and a collision energy of −35.0 eV. To also consider the possibility of condensation of the pyranoanthocyanin and ascorbic acid, the following single ion monitoring was also included: 675 *m*/*z* for 5-carboxypyranocyanidin-3-galactoside (517) + ascorbic acid (176) − H_2_O (18) and 673 *m*/*z* for dehydroscorbic acid (174).

#### 3.2.10. Statistical Analysis of Data

Data was organized for means and standard deviations using Microsoft Excel (Redmond, Washington, DC, USA). One-way ANOVA was performed for each treatment at all time points to determine if a significant change in CIE-L*c*h and maximum absorbance occurred. One-way ANOVA (two-tailed) was also performed for each pigment (control, 250, 500, 1000 mg/L AA) at each time point to determine if and when which CIE-L*c*h and maximum absorbance became significantly different from the control. Software used for ANOVA tests was SPSS (IBM, North Castle, NY, USA).

## 4. Conclusions

Cyanidin-3-galactoside degraded rapidly in the presence of ascorbic acid, followed by chokeberry extract. Other phenols in chokeberry extract likely played a protective role against AA-mediated pigment bleaching. The 5-Carboxypyranocyanidin-3-galactoside colored solution exhibited the smallest change in color (ΔE) and limited bleaching in response to ascorbic acid (for 1000 mg/L AA, ΔE of 5.2 versus 27.6 for cyanidin-3-galactoside). The interaction between PACN-AA resulted in the formation of three new chromophores, as revealed by HPLC. NMR work is underway to determine the site of reaction for PACN-AA as well as the ACN-AA reactivity. The fact that PACN, with the C4 position blocked, still exhibited limited bleaching further supports the hypothesis that C4 plays an important, but not singular, role in the AA-mediated bleaching of anthocyanins. The pyranoanthocyanin better maintained absorbance and color expression in the presence of AA, not as a result of 5-Carboxypyranocyandin-3-galactoside survival, but due to the formation of colored byproducts suspected at alternative sites.

## Figures and Tables

**Table 1:** Reaction rates and half-life (t_½_) of solutions colored with different pigments stored at 25 °C in the dark, modeled with first-order kinetics. Calculations are based on the changes in absorbance at the λ_vis-max_ of the solution over time.

Ascorbic Acid Level	Pigment	k (hour^−1^)	t_1/2_ (h)	R^2^
Control	Chokeberry Extract	8.08 × 10^4^	858	0.947
	Cyanidin-3-galactoside	1.27 × 10^3^	546	0.957
	5-Carboxypyranocyanidin-3-galactoside	7.08 × 10^4^	978	0.977
250 mg/L AA	Chokeberry Extract	1.02 × 10^2^	68	0.991
	Cyanidin-3-galactoside	3.18 × 10^2^	22	0.996
	5-Carboxypyranocyanidin-3-galactoside	2.69 × 10^3^	258	0.992
500 mg/L AA	Chokeberry Extract	1.60 × 10^2^	43	0.991
	Cyanidin-3-galactoside	5.90 × 10^2^	12	0.998
	5-Carboxypyranocyanidin-3-galactoside	4.61 × 10^3^	150	0.965
1000 mg/L AA	Chokeberry Extract	2.85 × 10^2^	24	0.999
	Cyanidin-3-galactoside	8.64 × 10^2^	8	0.996
	5-Carboxypyranocyanidin-3-galactoside	1.08 × 10^2^	64	0.998

**Table 2:** Days 0 and 5 colorimetric values (CIE-L*c*h*) and total color change (ΔE) of chokeberry, cyanidin-3-galactoside and 5-Carboxypyranocyanidin-3-galactoside for all AA levels over time. Numbers are means of three replications, followed by (standard deviations). ΔE: total color change of the solutions from day 0 to day 5. Values reported are means (*n* = 3) followed by standard deviation in parentheses.

Treatment		Lightness		Chroma		Hue Angle		ΔE
		Day 0	Day 5	Day 0	Day 5	Day 0	Day 5	Over 5 Days
Chokeberry Extract	Control	75.1 (0.5)	76.3 (0.5)	51.6 (0.8)	48.4 (1.2)	16.6 (0.5)	14.9 (0.8)	3.3 (0.4)
	250 mg/L AA	74.7 (0.2)	88.0 (0.2)	52.3 (0.4)	22.8 (0.3)	17.4 (0.2)	17.5 (0.5)	29.9 (0.4)
	500 mg/L AA	75.0 (0.3)	91.1 (0.6)	51.8 (0.6)	15.0 (0.4)	16.6 (0.3)	34.0 (3.1)	36.9 (0.2)
	1000 mg/L AA	74.4 (0.2)	92.7 (1.1)	52.9 (0.2)	13.3 (0.6)	17.6 (0.2)	53.8 (3.1)	42.0 (2.2)
Cyanidin-3-galactoside	Control	77.3 (0.1)	79.4 (0.8)	51.2 (0.2)	46.5 (2.1)	18.6 (0.1)	15.9 (1.0)	2.6 (1.1)
	250 mg/L AA	77.5 (0.2)	93.2 (0.3)	50.8 (0.2)	13.9 (0.7)	18.7 (0.1)	21.9 (1.2)	19.3 (0.3)
	500 mg/L AA	77.0 (0.1)	94.4 (0.6)	51.8 (0.0)	10.5 (0.4)	18.8 (0.0)	51.1 (2.7)	23.6 (0.2)
	1000 mg/L AA	77.2 (0.3)	94.5 (0.7)	50.9 (0.1)	13.7 (1.8)	18.7 (0.1)	77.5 (4.5)	27.6 (0.7)
5-Carboxypyranocyanidin-3-galactoside	Control	82.2 (0.2)	82.8 (0.6)	54.3 (0.7)	49.9 (1.3)	50.0 (0.2)	47.5 (0.6)	2.1 (0.4)
	250 mg/L AA	81.9 (0.1)	82.8 (0.3)	55.2 (0.4)	43.2 (0.2)	50.7 (0.2)	46.9 (0.3)	4.5 (0.2)
	500 mg/L AA	82.1 (0.3)	83.6 (0.4)	54.7 (0.3)	39.2 (0.3)	50.4 (0.1)	48.3 (0.4)	5.0 (0.2)
	1000 mg/L AA	81.7 (0.5)	83.7 (0.2)	55.6 (0.7)	36.2 (0.3)	51.0 (0.1)	57.1 (0.7)	5.2 (0.5)
